# Kinesin-5 is a microtubule polymerase

**DOI:** 10.1038/ncomms9160

**Published:** 2015-10-06

**Authors:** Yalei Chen, William O Hancock

**Affiliations:** 1 Department of Biomedical Engineering, The Pennsylvania State University, University Park, Pennsylvania 16802, USA; 2 Interdisciplinary Graduate Degree Program in Cell and Developmental Biology, Huck Institutes of the Life Sciences, The Pennsylvania State University, University Park, Pennsylvania 16802, USA

## Abstract

Kinesin-5 slides antiparallel microtubules during spindle assembly, and regulates the branching of growing axons. Besides the mechanical activities enabled by its tetrameric configuration, the specific motor properties of kinesin-5 that underlie its cellular function remain unclear. Here by engineering a stable kinesin-5 dimer and reconstituting microtubule dynamics *in vitro*, we demonstrate that kinesin-5 promotes microtubule polymerization by increasing the growth rate and decreasing the catastrophe frequency. Strikingly, microtubules growing in the presence of kinesin-5 have curved plus ends, suggesting that the motor stabilizes growing protofilaments. Single-molecule fluorescence experiments reveal that kinesin-5 remains bound to the plus ends of static microtubules for 7 s, and tracks growing microtubule plus ends in a manner dependent on its processivity. We propose that kinesin-5 pauses at microtubule plus ends and enhances polymerization by stabilizing longitudinal tubulin–tubulin interactions, and that these activities underlie the ability kinesin-5 to slide and stabilize microtubule bundles in cells.

## Abstract (web summary)

Kinesin-5 is a tetrameric motor that slides antiparallel microtubules during mitotic spindle assembly. Chen and Hancock show that this motor also promotes microtubule assembly by stabilising protofilaments at growing plus ends, which results in the formation of banana peel-like structures.

## Graphical abstract

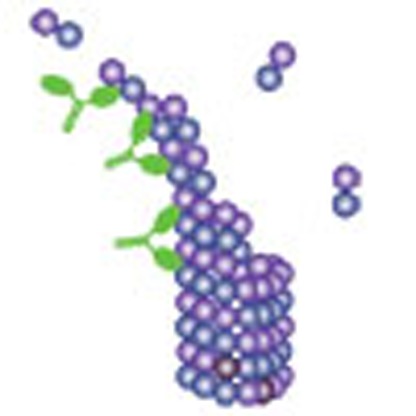

Kinesin-5 motors are responsible for separating duplicated poles and maintaining proper spindle bipolarity in dividing cells^1^,^2^,^3^,^4^, and regulating axonal growth and branching in developing neurons^5^,^6^. They carry out these activities by the virtue of their antiparallel tetrameric structure, which allows them to bundle and slide parallel and antiparallel microtubules^7^,^8^. Kinesin-5 motors generally move slower and are less processive than conventional transport kinesins, but the specific mechanochemical properties of the motors that underlie their cellular roles are not fully delineated^9^. Importantly, replacing the motor domains in a kinesin-5 tetramer with those from other kinesins leads to spindle collapse in a reconstituted system, suggesting that the tetrameric structure alone is not sufficient for kinesin-5 function^10^. Because of their important role in mitosis, kinesin-5 motors are targets for potential anticancer drugs, emphasizing the importance of a more thorough understanding of the specific mechanochemical properties that underlie their cellular functions.

A number of kinesins have been found to accumulate at microtubule plus ends and affect microtubule dynamics. The yeast kinesin-8, Kip3p, accumulates at plus ends and depolymerizes microtubules, and kinesin-13 motors are also potent depolymerizers^11^,^12^,^13^,^14^,^15^. This depolymerase activity is thought to result from the motors actively removing tubulin from the microtubule end, which can then trigger a catastrophe event^11^,^16^. The kinesin-7 motor CENP-E binds to the plus ends of both growing and shrinking microtubules, and promotes plus-end elongation of stabilized microtubules *in vitro*^17^,^18^. Other kinesins such as the kinesin-4, KIF4, and the mammalian kinesin-8, KIF18A, localize to microtubule plus ends and suppress microtubule dynamics^19^,^20^,^21^,^22^,^23^,^24^. In contrast to the depolymerizing kinesins, the mechanisms underlying these activities are less clear, but are thought to involve motors binding through one or both motor domains to the microtubule plus end and slowing addition and/or dissociation of tubulin subunits. A number of kinesins that bind microtubule plus ends require their tail domain for stable plus-end attachment, suggesting a model in which plus-end accumulation involves contributions from both the motor and tail domains^11^,^12^,^18^. End binding by dimeric motors lacking any secondary microtubule binding sites has been observed^11^; however, the unique features of the chemomechanical cycle that enable this behaviour are not clear.

From studies of spindle dynamics in yeast there are conflicting reports for the effects of kinesin-5 motors on microtubule polymerization. Gardner *et al*.^25^ found that knocking out the yeast kinesin-5 Cin8 resulted in longer kinetochore and astral microtubules, suggesting that the motor acts as a depolymerase^26^. In contrast, Fridman *et al*.^27^ found that knocking down Kip1p increased microtubule dynamics, suggesting that the motor acts as a microtubule stabilizer. Notably, both the studies found that kinesin-5 motors localized to microtubule plus ends, which is presumably a prerequisite for altering plus-end dynamics. Consistent with this localization, it was shown in *in vitro* assays that following antiparallel microtubule sliding mediated by *Xenopus* kinesin-5 tetramers, the plus ends of the two microtubules remained associated for a number of seconds, consistent with the motors pausing at the plus ends before dissociating^7^. What is not clear is whether this end association is mediated by the motor domains or the C-terminal tail, which also can bind microtubules^8^,^28^.

In the present work, we use an engineered kinesin-5 dimer to address the following questions: how does kinesin-5 interact with microtubule plus ends, and what effect does kinesin-5 have on microtubule dynamics? Removing the tetramerization domain and C-terminal tail allows us to focus solely on the activity of the motor domains, and enables comparison of the kinesin-5 chemomechanical mechanism with that of other non-mitotic kinesins. We find that kinesin-5 dimers pause at microtubule plus ends for multiple seconds, accelerate the microtubule growth rate and track with the growing microtubule plus ends. Thus, kinesin-5 shares some of the same biochemical activities of the microtubule polymerase XMAP215, but achieves it through a different mechanism. These end-binding and polymerase activities may be essential for the function of wild-type kinesin-5 in the mitotic spindle.

## Results

### Kinesin-5 does not depolymerize GMPCPP microtubules

**Figure 1:**
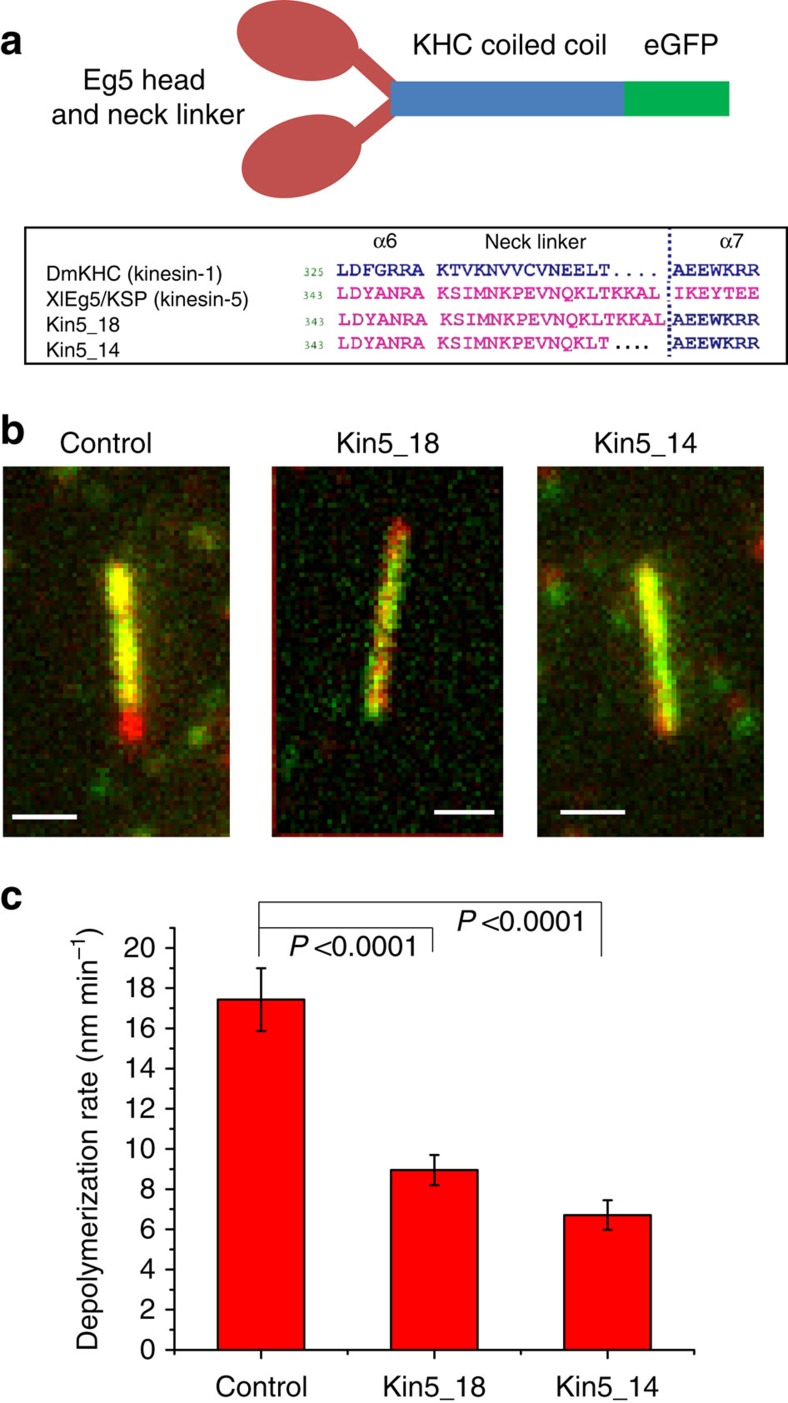
Kinesin-5 stabilizes GMPCPP microtubules. (**a**) Diagram of dimeric kinesin-5 constructs Kin5_18 and Kin5_14 (ref. 33). (**b**,**c**) Kinesin-5 slows depolymerization of GMPCPP microtubules. Surface-immobilized GMPCPP microtubules were incubated in the presence or absence of motors, as indicated. (**b**) Superimposed before/after images with initial microtubule image in red and microtubule after 20 min in green. Scale bar, 1 μm for all. (**c**) Mean depolymerization rate in the absence and presence of 20 nM Kin5_14 or 25 nM Kin5_18 over a 20-min interval. *P* values are from two sample *t*-tests.

To test for depolymerase activity, we investigated the influence of a dimeric kinesin-5 construct on the stability of GMPCPP-stabilized microtubules. In the absence of free tubulin, GMPCPP microtubules depolymerize at a slow rate^29^, and both the kinesin-8 Kip3p^11^,^12^,^30^ and the kinesin-13 MCAK^15^,^31^ have been shown to substantially accelerate the shortening rate. To eliminate potential regulation and microtubule binding by the C-terminal domain of kinesin-5, experiments used a stable kinesin-5 dimer that was previously shown to have comparable motor properties to full-length kinesin-5, and was made by fusing the motor domain and neck-linker region of *Xenopus laevis* Eg5 to the proximal coiled coil of *Drosophila* conventional heavy-chain (KHC) (Fig. 1a; see Methods)^32^,^33^. This dimer with its native 18 amino acids neck linker (Kin5_18) is minimally processive with a run length of 0.33±0.03 μm (mean±s.e.m.), and an identical construct with its neck linker shortened to 14 residues (Kin5_14) had a run length of 1.02±0.12 μm (mean±s.e.m.; Supplementary Fig. 1)^33^. This more processive Kin5_14 construct was used in a subset of the experiments because its longer run length facilitated mechanistic analysis. In the absence of kinesin-5, surface-immobilized GMPCPP microtubules depolymerize slowly at an average speed of 17.4±1.6 nm min^−1^ (mean±s.e.m., *n*=26, Fig. 1b,c), as seen previously^11^. In the presence of either Kin5_14 or Kin5_18, the depolymerization rate was slowed by approximately twofold (Fig. 1b,c). Thus, kinesin-5 does not act as a depolymerase under these conditions, and instead promotes stabilization of GMPCPP microtubules.

### Kinesin-5 promotes polymerization of dynamic microtubules

**Figure 2:**
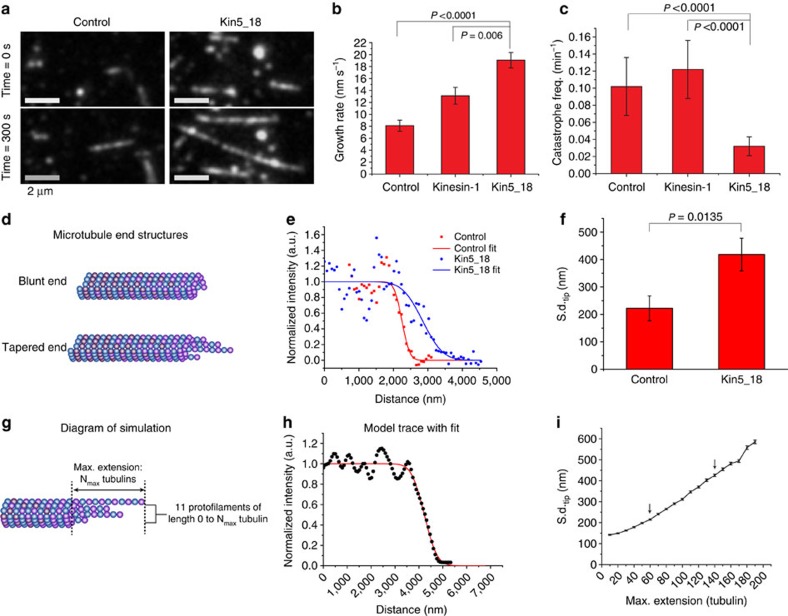
Kinesin-5 acts as a microtubule polymerase. (**a**) Sample images of microtubules extended from GMPCPP seeds for 5 min in 10 μM free tubulin in the presence and absence of 20 nM Kin5_18. Scale bar, 2 μm (for all). (**b**,**c**) Microtubule growth rate and catastrophe frequency in the absence of motor (control), or in the presence of 100 nM kinesin-1 or 20 nM Kin5_18, showing that kinesin-5 causes a significant increase in the growth rate and significant decrease in the catastrophe frequency. (**d**) Diagram of proposed plus-end structures for microtubules growing under different conditions. (**e**) Fluorescence intensity profile of growing microtubule plus ends. Line scans (points) of fluorescence intensity along microtubules were each normalized relative to background, and then fit by error functions (lines). (**f**) Tip s.d. values obtained from error function fits in **e**, showing that Kin5_18 causes significant increase plus-end tapering. (**g**) Diagram of simulation used to predict fluorescence profile of tapered microtubule plus ends. To simulate the end tapering of a 13 protofilament microtubule, the longest protofilament was fixed at *N*_max_ tubulin subunits longer than the shortest protofilament, and the lengths of the remaining 11 protofilaments were randomly selected from 0 to *N*_max_. The dye density (1 dye per 20 tubulin dimers) matched the experimental conditions. (**h**) An exemplary intensity profile and corresponding fit from a simulation with *N*_max_=150 tubulin. (**i**) Plot of tip s.d. versus *N*_max_, showing that s.d._tip_ of 222 for the control corresponds to tapering over ∼60 tubulin subunits and s.d._tip_ of 418 nm for the motor case corresponds to tapering over 140 tubulin. All error bars in figure represent s.e.m.

To investigate the impact of kinesin-5 on dynamic microtubules, surface-immobilized GMPCPP seeds were extended using cy5 tubulin, and the resulting microtubule dynamics was observed by total internal reflection fluorescence (TIRF) microscopy. The first clear observation was that following a 5-min incubation, microtubules polymerized in the presence of Kin5_18 were longer than control microtubules (Fig. 2a). We next quantified the microtubule dynamic instability parameters of growth rate, shrinkage rate, catastrophe frequency and rescue frequency at 10 μM Cy5 tubulin and 30 °C. The addition of 30 nM Kin5_18 increased the growth rate more than twofold, from 8.1±0.9 to 19.1±1.3 nm s^−1^ (mean±s.e.m., *n*=19 and 21, respectively; Fig. 2b). Furthermore, Kin5_18 reduced the catastrophe frequency by a factor of three from 0.102±0.034 to 0.032±0.011 min^−1^ (mean±s.d., *n*=9 for each; Fig. 2c). In contrast, there was no statistically significant change in the rescue frequency (Supplementary Fig. 2a) and the shrinkage rate increased in presence of Kin5_18, from 43.7±5.5 to 79.2±10.2 nm s^−1^ (mean±s.e.m., *n*=9 for both; Supplementary Fig. 2b), but as shown in Supplementary Table 1, this change in the shortening rate had a negligible effect on the net microtubule growth rate. As a control, the same experiments were repeated in 100 nM kinesin-1, and there was a slight increase in the microtubule growth rate but no change in the catastrophe frequency (Fig. 2b,c). Similarly, there was no effect on the shortening rate, or rescue frequency (Supplementary Fig. 2b).

### Kinesin-5 enhances microtubule plus-end tapering

Microtubule plus ends are thought to be stabilized by a cap of GTP tubulin, with catastrophe resulting from loss of this GTP cap. Due to stochasticity in the addition of tubulin dimers and varying dimer dissociation rates based on stabilizing lateral and longitudinal tubulin–tubulin interactions, different protofilaments at the growing microtubule plus end will extend at different speeds^34^,^35^. Fluorescence experiments and stochastic simulations showed that longer periods of growth and faster growth rates (resulting from increased free tubulin concentrations) resulted in microtubule plus ends becoming more tapered^36^ (Fig. 2d). Furthermore, increased tapering was correlated with elevated catastrophe frequencies, providing a link between microtubule dynamics and microtubule plus-end structures^36^. To understand its influence on microtubule dynamics, we quantified the degree of plus-end tapering of microtubules polymerized in the presence and absence of kinesin-5 by performing line scans and fitting the fall of fluorescence intensity at the tip by an error function, as carried out previously^36^,^37^,^38^ (Fig. 2e; see Methods). The s.d. from the fit represents the degree of tapering at the end, with a larger tip s.d. (s.d._tip_) denoting a more tapered end. For control microtubules, s.d._tip_ was 222±45 nm, and in the presence of Kin5_18 it increased to 418±59 nm (mean±s.e.m., *n*=19 for both; Fig. 2f). This result demonstrates that kinesin-5 alters the structure of growing microtubule plus ends.

Ultimately, the point spread function of the microscope limits the ability to determine the degree of plus-end tapering. Hence, to better quantify the degree of tapering, we simulated different plus-end structures (Fig. 2g; see Methods), convolved the structures with the measured point spread function (309 nm full-width half-maximum, see Methods), and compared the resulting error function fits with the experimental results (Fig. 2h,i). The 222 nm s.d._tip_ of the control corresponds to tapering over a length of ∼60 tubulin dimers, and the 418 nm s.d._tip_ of the motor sample correlates to tapering over ∼140 tubulin dimers. Thus, the results are consistent with kinesin-5 more than doubling the length of protofilament extensions at the plus ends of growing microtubules.

### Kinesin-5 generates diverse structures at growing plus ends

**Figure 3:**
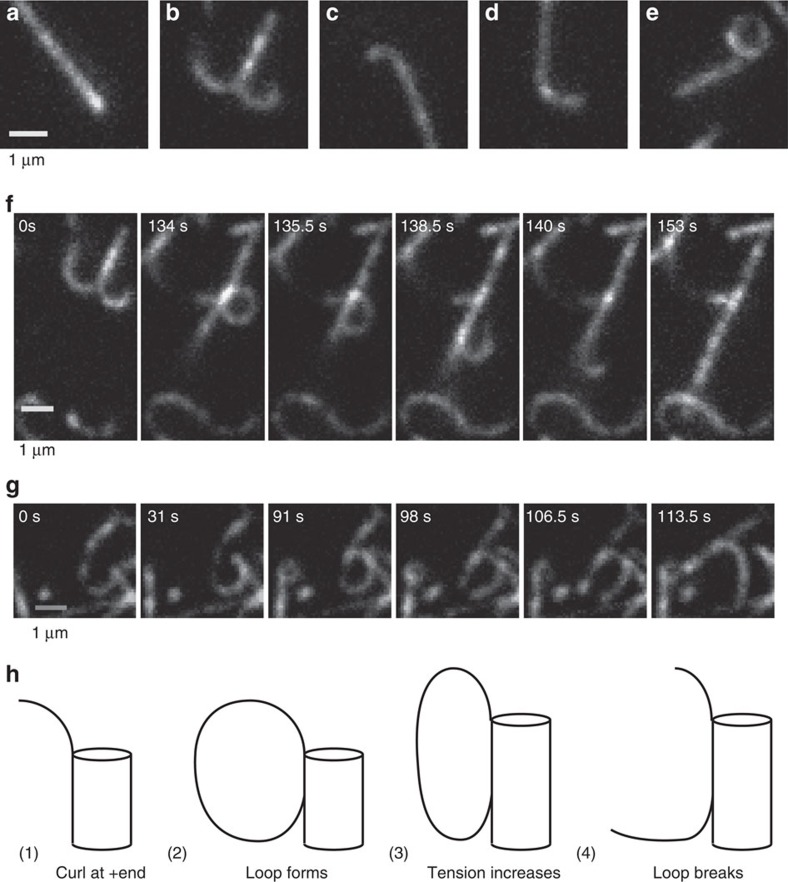
Diverse plus-end structures of microtubules grown in the presence of kinesin-5. (**a**–**e**) Images of microtubules growing in the presence of 10 μM tubulin and 30 nM Kin5_18GFP, showing straight (**a**), bifurcated (**b**), curved (**c**,**d**) and looped (**e**) plus ends. Tubulin was unlabelled and fluorescence was solely due to the Kin5_18GFP. (**f**) Annealing of a ‘banana peel'. In this case, the microtubule initially grows with a bifurcated plus end, one side straightens at 134 s, and the second side straightens and anneals between 135.5 and 140 s. For full sequence, see Supplementary Movie 2. (**g**,**h**) Images and diagram of protofilaments curling to make a loop and then breaking. The curled plus end at 0 s grows to form a closed loop at 91 s, which breaks at 98 s. For full sequence, see Supplementary Movie 3.

To understand the mechanisms by which kinesin-5 affects microtubule dynamics, we observed the dynamics of green fluorescent protein (GFP)-tagged kinesin-5 at the growing plus ends of microtubules polymerized from unlabelled tubulin, a strategy that avoids the high backgrounds inherent in using fluorescent tubulin. A range of striking and diverse plus-end structures were observed (Fig. 3a–e), which were not seen in control experiments with kinesin-1-GFP (Supplementary Movie 1). A subset of microtubules grew with long curved plus ends that in some cases bifurcated (Fig. 3b), and in other cases formed closed rings (Fig. 3e). Shown in Fig. 3f (Supplementary Movie 2) is a microtubule that grows with a bifurcated plus end, reminiscent of the ‘banana peel' structures proposed for shrinking microtubule plus ends^39^. As the microtubule grows, one branch straightens, followed by straightening and annealing of the second branch to form an apparently intact microtubule. This behaviour is consistent with kinesin-5 promoting the formation of curved bundles of protofilaments that are straightened on integration into the microtubule lattice. In another example, a curved plus-end extension apparently forms a closed loop that then breaks and is incorporated into the growing microtubule (Fig. 3g,h; Supplementary Movie 3).

The frequency of these curved plus-end structures was quantified by observing microtubules growing over 5-min windows and defining an event as a tip structure that is curved at least 90° from the microtubule axis (see Fig. 3a for examples). For microtubules polymerizing in the presence of kinesin-5, 72% of microtubules (34 out of 47) had curved tip structures, while no microtubules grown in the presence of kinesin-1 (0 out of 38) had curved tip structures.

### Kinesin-5 binds to plus ends of taxol-stabilized microtubules

**Figure 4:**
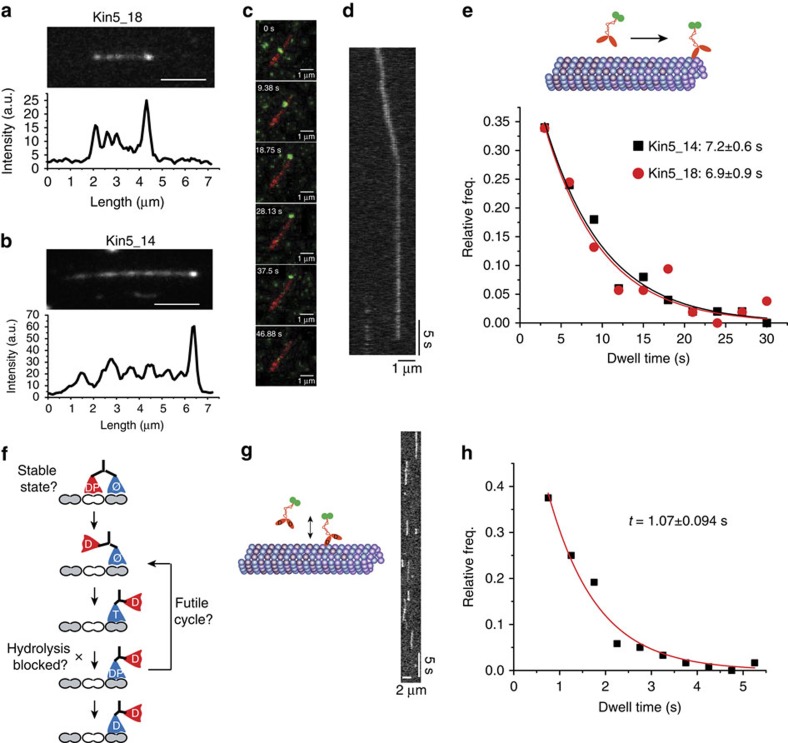
Kinesin-5 pauses at plus ends of taxol-stabilized microtubules. (**a**,**b**) Kin5_18GFP and Kin5_14GFP (5 and 3 nM, respectively, in 1 mM ATP) walking on and accumulating at plus ends of taxol-stabilized microtubules. (**c**,**d**) Sequence of images (**c**) and kymograph (**d**) at 25 pM motor concentration, showing a single Kin5_14GFP molecule walking along a taxol-stabilized microtubule and pausing at the plus end before dissociating. (**e**) Distribution of motor residence times at plus ends of taxol-stabilized microtubules, along with mean and s.e. of exponential fit. *N*=137 and 98 for Kin5_18 and Kin5_14, respectively. (**f**) Simplified hydrolysis cycle of a motor waiting at a microtubule plus end. Three possible explanations for the end-binding duration are that the motor binds in a two-head-bound configuration, the motor undergoing futile hydrolysis cycles, or ATP hydrolysis requires binding of the tethered head to the next tubulin on the lattice (see text for further details). (**g**) Diagram and kymograph of Kin5_18GFP landing on and dissociating from an immobilized microtubule in 1 mM ADP. (**h**) Distribution of motor binding durations in 1 mM ADP, along with mean and s.e. of exponential fit to the data, giving an average residence time that is considerably shorter than the dwell time at plus ends in ATP.

To explore kinesin-5 activity at microtubule plus ends, we used TIRF microscopy to observe interactions between kinesin-5 and immobilized taxol-stabilized microtubules. At low nM motor concentrations, streaming of motors along microtubules was observed (Fig. 4a,b), along with clear accumulation at plus ends, suggesting that motors walk to plus ends and pause there. To characterize these pause durations, motor concentrations were lowered to single-molecule levels (25 pM), where processive runs and plus-end pauses were clearly observed (Fig. 4c,d). Exponential fits to pause lifetime distributions gave mean pause durations of 7.2±0.6 and 7.0±0.9 s (mean±s.e.m.) for Kin5_14 and Kin5_18, respectively (Fig. 4e). Thus, when reaching the plus end, the motors bind there 40-fold longer than the normal 125-ms step duration, and the duration of end binding is independent of motor processivity.

On the basis of the standard kinesin hydrolysis cycle, in saturating ATP the motor should wait at the plus end either in a two-head-bound state, or in a one-head-bound state with ATP, ADP-Pi or ADP in the bound head^40^ (Fig. 4f). To test the hypothesis that kinesin-5 remains bound in the ADP state, the binding of Kin5_18GFP to the microtubule lattice at 1 mM ADP was measured. The mean duration was 1.07±0.09 s (mean±s.e.m., *n*=120, Fig. 4g,h), ruling out a model in which the motor waits in a weak-binding ADP state, and suggesting a model in which the motor at the plus end is undergoing futile hydrolysis cycles, is unable to hydrolyse ATP or remains bound in a two-head-bound state, possibly due to protofilament curvature at the end.

**Figure 5:**
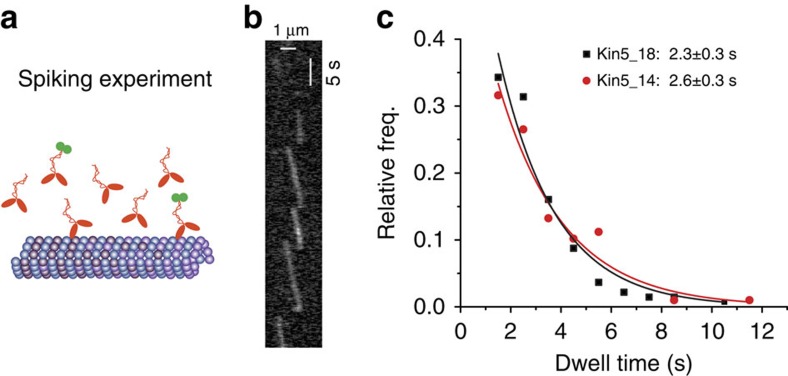
Plus-end pausing under crowded conditions. (**a**) Diagram of spiking experiments. (**b**) Kymograph of Kin5_14GFP walking to plus end of a taxol-stabilized microtubule in the presence of excess unlabelled motors. (**c**) Distribution of dwell times of motors at plus ends under crowded conditions, with exponential fits to data, resulting mean durations of 2.26s and 2.61 s for Kin5_18 and Kin5_14, respectively. Experimental conditions: 50 pM Kin5_14GFP plus 15 nM unlabelled Kin5_14, and 70 pM Kin5_18GFP plus 25 nM unlabelled Kin5_18.

Previous work on the microtubule depolymerizing kinesin-8 motor, Kip3p, showed that the arrival of new motors to the plus-end promoted motor dissociation and contributed to the depolymerase activity (the ‘bump-off' model)^11^. To measure kinesin-5 end durations under crowding conditions, a spiking experiment was carried out in which 70 pM of GFP-labelled motor was mixed with 15 nM unlabelled motor and the single-molecule binding durations at plus ends of taxol-stabilized microtubules was assessed (Fig. 5a,b). Under these crowded conditions, the end duration of Kin5_18GFP was reduced to 2.26±0.30 s, and the end duration of Kin5_14GFP was decreased to 2.61±0.29 s (mean±s.e.m.; Fig. 5c). These findings suggest that direct interactions between kinesin-5 motors at microtubule plus ends accelerate motor dissociation, but also show that even under these crowded conditions the waiting time at the plus end is still ∼20-fold longer than the normal stepping rate.

### Kinesin-5 tracks plus ends of growing microtubules

**Figure 6:**
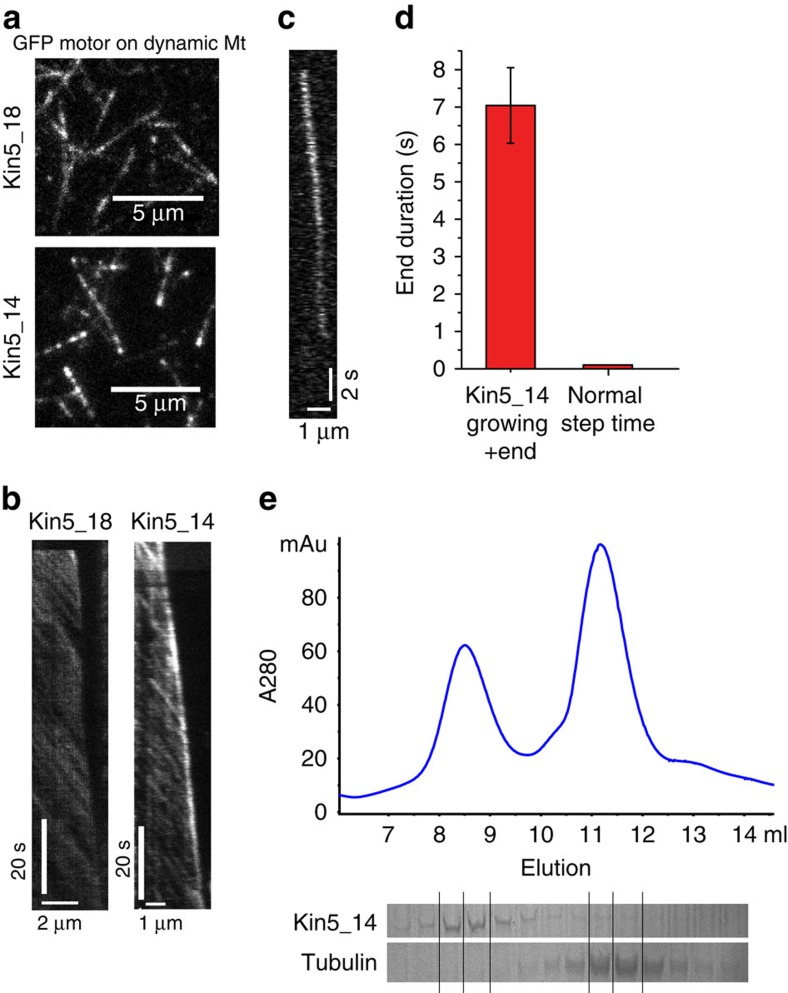
Kinesin-5 tracks growing microtubule plus ends. (**a**,**b**) Images and kymographs of Kin5_18GFP and Kin5_14GFP labelling plus ends of growing microtubules. Conditions were 20 nM Kin5_14GFP or 30 nM Kin5_18GFP, 20 μM tubulin and 1 mM ATP. (**c**) Single-molecule kymograph. A total of 50 pM Kin5_14GFP and 20 μM tubulin were used. (**d**) Mean duration of kinesin-5 residence at plus ends, compared with motor step duration on microtubule lattice. (**e**) Gel filtration of 3 μM Kin5_14 and 20 μM tubulin in 1 mM ATP shows distinct peaks of tubulin and motors and no evidence of motor–tubulin complex.

Plus-tip-interacting proteins preferentially bind to growing microtubule plus ends and in some cases modulate microtubule dynamics^41^. To observe tip-tracking activity of kinesin-5, microtubules were polymerized in 20 μM tubulin in the presence of GFP-labelled motors. While only occasional plus-end accumulation was observed for Kin5_18, the more processive Kin5_14 consistently labelled growing microtubule plus ends (Fig. 6a,b), demonstrating that kinesin-5 has plus-tip-interacting protein activity. At single-molecule concentrations (50 pM), the mean residence time of Kin5_14 at dynamic plus ends was 7.04±1.01 s (mean±s.e.m. of fit; Fig. 6c,d), similar to the mean residence time at taxol-stabilized microtubule plus ends (Fig. 4h). Thus, the more processive Kin5_14 pauses at both static and dynamic plus ends, while the less processive Kin5_18 pauses at static plus ends but does not detectably pause at dynamically growing plus ends at these experimental conditions. A model to account for this discrepancy is presented in Supplementary Fig. 3.

### Kinesin-5 does not measurably bind free tubulin in solution

To better understand the kinesin-5 polymerase mechanism, we investigated whether kinesin-5 binds free tubulin in solution. Like kinesin-5, XMAP215 enhances polymerization, plus-tip tracks on growing microtubules, and binds plus ends for several seconds. XMAP215 achieves this activity through a series of linked TOG domains that bind free tubulin and facilitate its integration into the growing microtubule plus end^42^,^43^. Thus, one possible mechanism for the polymerase activity of kinesin-5 is that the motor waits at the microtubule plus end with one-head bound to the terminal tubulin dimer, while the second head binds a free tubulin from solution and facilitates its integration into the growing protofilament. To test the plausibility of this model, we tested for a direct motor–tubulin interaction by mixing 3 μM Kin5_14 dimers with 20 μM free tubulin in 1 mM ATP and separating them by gel filtration chromatography. As seen in Fig. 6e, only two peaks corresponding to kinesin and tubulin were seen, with no evidence for a complex. While these results do not rule out any interactions between kinesin-5 and free tubulin, they do demonstrate that the *K*_D_ for the motor binding to free tubulin is considerably above the tubulin concentrations used in these experiments. Thus, they provide a strong argument against a model in which kinesin-5 enhances polymerization by binding free tubulin in solution and facilitating its incorporation into the growing microtubule.

## Discussion

Kinesin-5 carries out the essential cellular functions of sliding apart antiparallel microtubules and maintaining outward-directed forces in the mitotic spindle. We show here that when kinesin-5 reaches a microtubule plus end, it remains bound there, and that this activity enhances microtubule polymerization. Both of these activities are important in cells—end binding will enhance the ability of tetrameric motors to push apart antiparallel microtubules, and polymerase activity will help to ensure that the regions of microtubule overlap necessary for kinesin-5 to generate outward-directed forces are maintained.

**Figure 7:**
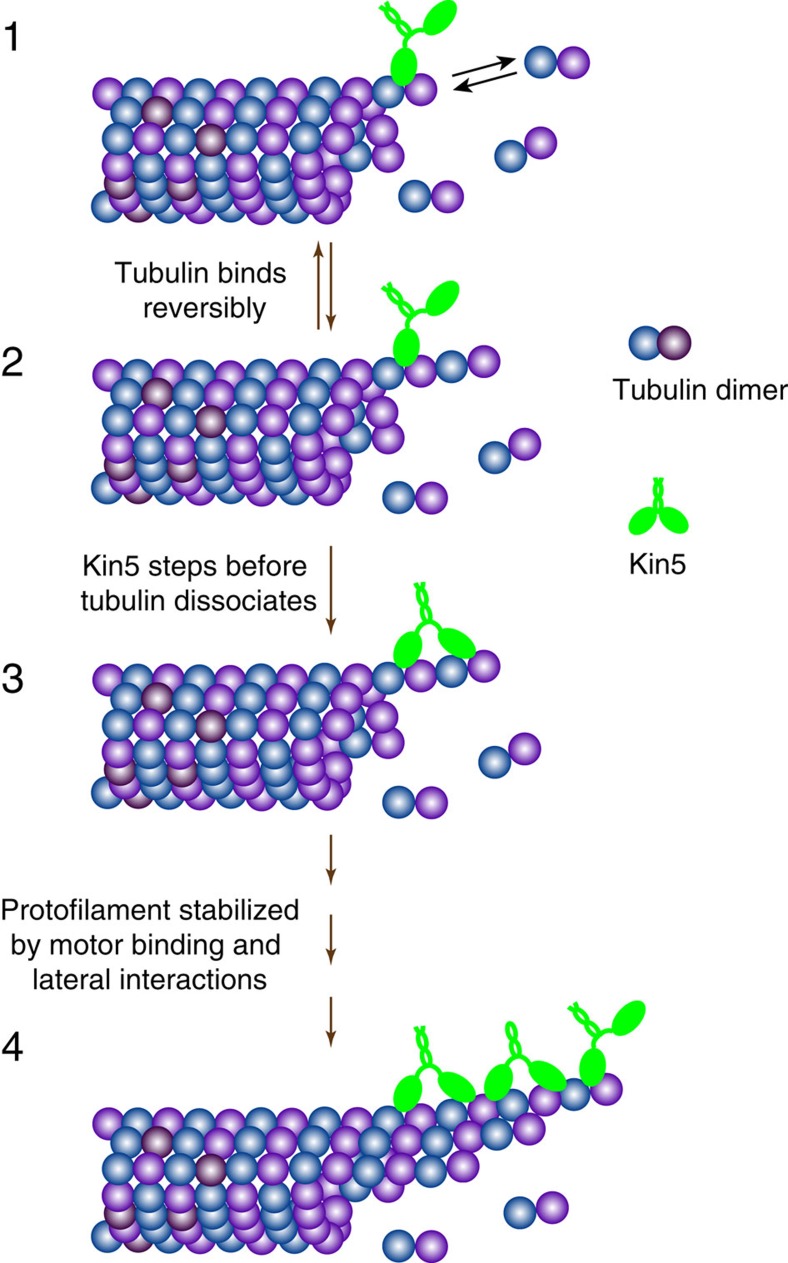
Proposed kinesin-5 polymerase mechanism. Motors walk to the plus end of protofilaments where tubulin subunits are being rapidly and reversibly exchanged (state 1). If the motor steps before a newly added tubulin subunit dissociates, then it stabilizes the longitudinal tubulin–tubulin interactions and slows the tubulin off-rate (state 3). This stabilization enhances the microtubule growth rate and suppresses the catastrophe frequency. Over time, growing protofilaments will be stabilized by lateral interactions with adjacent protofilaments (state 4), leading to long and stable protofilament bundles. Following catastrophe, the rapid depolymerization rates preclude plus-end motor accumulation and hence are expected to have little effect on the depolymerization rate.

Our results converge on a model in which kinesin-5 motors walk along a microtubule, pause at the terminal tubulin subunit of a protofilament, and promote incorporation of the next tubulin dimer (Fig. 7). While this model qualitatively accounts for the results, there are two principal questions. First, why does the motor pause instead of walking off the end of the protofilament? Second, how do the end-bound motors enhance polymerization? These questions are addressed in sequence below.

During their hydrolysis cycle, kinesins cycle between high microtubule affinity (ATP-bound and no nucleotide) and lower microtubule affinity (ADP-Pi and ADP) states^40^. We find that dimeric kinesin-5 steps at ∼8 steps per second (125 ms per step), binds to the microtubule lattice in its weak binding ADP state for 1 s (Fig. 4h), and in saturating ATP waits at microtubule plus ends for 7 s before dissociating (Fig. 4e). The fact that end binding is observed on both dynamic and taxol-stabilized microtubules means that if motors are binding to some structural feature present at plus ends, then this feature is present in both static and dynamic microtubules. The fact that shortening the kinesin-5 neck linker domain enhanced processivity without changing the end-binding duration suggests that the mechanisms regulating end binding are distinct from the mechanisms regulating motor processivity. Also, the 1-s binding duration in ADP argues against a mechanism in which the motor simply waits at the plus end in its low-affinity ADP state.

There are three plausible models that explain this plus-end pausing. The first possibility is that, during its stepping cycle, ATP hydrolysis in the bound head requires binding of the tethered head to the next binding site (Fig. 4f). This mechanism would ensure that the bound head at the end of a protofilament would reside in a no-nucleotide or ATP-bound state, both of which are high affinity. However, at present there is no biochemical support for this hypothesis. A second possibility is that end-bound motors carry out futile hydrolysis cycles, and the hydrolysis and product release rates are such that the motor spends a large fraction of its hydrolysis cycle in high microtubule affinity states. The suggestion that ATP hydrolysis is limiting for Eg5 is consistent with this model^44^, and if the motor spent 1/7th of its cycle in the ADP/ADP-Pi state and this state has an off-rate of 1 s^−1^, this could potentially explain the 1/7 s^−1^ off-rate. The third possibility is structural—if the terminal tubulin subunit in each protofilament is bent outward (analogous to ‘breathing' at the ends of duplex DNA strands), then the motor could bind to the last two tubulin subunits in a minimally strained two-head-bound state. In this model, a forward step would be triggered by binding of a new tubulin subunit and the resulting straightening of the protofilament. All three of these models are consistent with existing data.

Microtubule polymerization involves reversible binding of GTP tubulin dimers to protofilament ends via weak longitudinal tubulin–tubulin interactions, followed by stabilization in the lattice through lateral interactions with adjacent protofilaments^45^,^46^. Because the initial binding of tubulin to the protofilament end is thought to be reversible on rapid timescales^38^, together with the fact that kinesin-5 does not measurably bind free tubulin (Fig. 6d), the enhancement of polymerization is best explained by a mechanism in which the motor stabilizes the incoming tubulin by bridging it to the protofilament end, thus strengthening the weak longitudinal interactions until stabilizing lateral interactions can be established (Fig. 7). By slowing the rapid off-rate, this mechanism would accelerate the microtubule growth rate, and if this acceleration resulted in a larger GTP cap, then it would also be predicted to suppress the catastrophe frequency. In contrast, during a shrinking event any end-bound motors will dissociate along with the dissociating tubulin, and neither an effect on the shrinking rate nor an effect on the rescue frequency would be predicted (see Supplementary Table 1 for further discussion of depolymerization rates).

Kinesin-5 had no effect on the rescue frequency, but it did increase the shortening rate of dynamic microtubules (Supplementary Fig. 2). However, because motors do not accumulate at the plus ends of shrinking microtubules and because the rapid loss of GDP tubulin dimers from shrinking microtubules differs both kinetically and energetically from the addition of GTP tubulin dimers to growing microtubules, we consider the effects of motors on the shortening rate to be mechanistically distinct from effects on microtubule growth. The mechanism is not clear, but we hypothesize that the increased shortening rate in the presence of kinesin-5 is a secondary effect that results from defects in the lattice that are introduced due to the accelerated growth kinetics or the resolution of the diverse curved end structures into straight filaments when microtubules are polymerized in the presence of kinesin-5. While the finding that kinesin-5 increases the shortening rate of dynamic microtubules appears in conflict with their apparent stabilization of GMPCPP microtubules (Fig. 1b,c), the difference can be explained by the slow depolymerization rate of GMPCPP microtubules (>100-fold slower than depolymerizing dynamic microtubules), which allows motors to accumulate at their plus ends and exert a stabilizing influence there. As a final point, because microtubules grown in the presence of kinesin-5 spend most of their time in the growth phase, the dynamics of the shortening phase have a minimal effect on the overall microtubule growth rate (Supplementary Table 1).

One novel phenomenon, not previously observed for other motors or MAPs, was the diverse plus-end structures observed for microtubules polymerized in the presence of kinesin-5 (Fig. 3). Using cryoEM, Cretien *et al*. previously saw curved structures at the ends microtubules growing in high concentrations of free tubulin, but mean lengths were <300 nm (although very rare extensions of up to 2 μm were noted)^39^. In contrast, end structures observed here were microns long and in some cases made complete circles (Fig. 4). Furthermore, they were observed to ‘zipper up' and close into straight microtubules. What are these structures? One possibility is that they are single protofilaments extending from the microtubule. However, even with stabilization by motors it is unlikely that longitudinal interactions alone would support single protofilaments that are microns long. Furthermore, depolymerizing protofilaments containing GDP tubulin have very tight radii of curvature (tens of nanometre at most)^39^, in contrast to the gradual curvatures observed here (order of 1-μm radii of curvature; see Fig. 3). Our tentative conclusion is that these curved extensions represent bundles of protofilaments having rigidities somewhere between a flexible protofilament and a stiff microtubule, and the observed ‘zippering' represents closing of the protofilament bundles into a cylindrical microtubule.

One way to understand the polymerase mechanism of kinesin-5 is to compare it with XMAP215, a polymerase consisting of a linked series of TOG domains that bind free tubulin and promote microtubule polymerization. XMAP215 and kinesin-5 are similar in that: (1) they accelerate microtubule growth rates; (2) individual molecules follow growing microtubule plus ends; and (3) they diffuse along the microtubule lattice^42^,^47^,^48^,^49^. However, they differ in that: (1) XMAP215 binds preferentially to free tubulin in the bent conformation^42^,^47^, while kinesin-5 binds preferentially to tubulin in the microtubule lattice; (2) XMAP215 accelerates the depolymerization of GMPCPP microtubules^42^,^50^, while kinesin-5 stabilizes them; and (3) XMAP215 is proposed to stabilize lateral tubulin–tubulin interactions^47^, while we propose that kinesin-5 stabilizes longitudinal tubulin–tubulin interactions. Thus, these two microtubule polymerases appear to achieve similar effects using different mechanisms.

The observed microtubule polymerase and end-binding activities are consistent with the role of kinesin-5 in mitosis. Kinesin-5 tetramers are essential in most cells for separating the duplicated poles to form a bipolar spindle and for generating outward forces necessary for spindle maintenance^1^,^2^,^51^,^52^. During antiparallel microtubule sliding, end pausing by one pair of dimeric heads will allow more time for the second pair of heads to generate pushing forces, and by enhancing the growth rate and inhibiting catastrophe, the motor will maximize the length of microtubule track on which it can walk. This polymerase activity also provides a potential explanation for the poleward microtubule flux observed in *Drosophila* embryos, *Xenopus* egg extracts and other systems^53^,^54^,^55^. During metaphase and anaphase A, it has been shown that kinesin-13 motors are involved in minus-end depolymerization at the poles^56^, and, until now, the arrest of poleward flux and eventual spindle collapse that result from inhibiting kinesin-5 were thought to result from blocking the force-generating capacity of the motor^52^,^53^,^54^,^55^,^57^. On the basis of the present findings, we hypothesize that kinesin-5 polymerase activity contributes to the net polymerization at the equator that is necessary to achieve poleward flux, and that this polymerization activity also explains why spindle collapse in kinesin-5-depleted *Xenopus* extracts cannot be rescued by modified kinesin-5 tetramers in which the heads are replaced by kinesin-1 motor domains^10^. The present work motivates experiments to test the influence of kinesin-5 on microtubule dynamics in dividing cells and the role that this polymerase activity plays in formation and maintenance of the mitotic spindle.

## Methods

### TIRF microscopy assay

Motors were bacterially expressed and purified, and quantified by GFP absorbance as previously described^33^. All experiments were carried out in BRB80 buffer (80 mM K-Pipes, 1 mM EGTA, 1 mM MgCl_2_, pH=6.8). Coverslips were cleaned in piranha, treated with 0.5% octadecyltrichlorosilane in toluene for 1 h, rinsed with toluene and assembled into flow cells as previously described^58^. Flow cells were incubated with 0.5 mg ml^−1^ neutravidin, followed by 5% Pluronic F108 in double distilled H_2_0 to block the surface. Cy5 tubulin, biotinylated tubulin and unlabelled tubulin at a ratio of 3:1:12 and a total concentration of 20 μM were polymerized at 37 °C for 30 min in the presence of 0.25 mM GMPCPP, and these GMPCPP Cy5-biotin microtubules were introduced in the presence of 1 mg ml^−1^ casein for 5 min. The surface-immobilized microtubules were then extended by introducing unlabelled free tubulin (10 μM unless otherwise specified) at 30 °C. To observe Cy5 microtubule growth, 0.5 μM Cy5 tubulin was mixed with unlabelled tubulin and antifade as previously described^58^. For microtubule depolymerization experiments, the laser was shuttered for most of the recording and focus was checked every 3–4 min.

### Gel filtration

A 500 μl sample of 3 μM Kin5_14 and 20 μM of unlabelled tubulin in BRB80 supplemented with 1 mM ATP was incubated at room temperature for 5 min before loading on to a Superdex 200 10/300 GL column (GE Healthcare) pre-equilibrated in BRB80 plus 1 mM ATP. Absorbance was monitored at 280 nm and 0.5 ml fractions collected and fractions analysed by SDS–polyacrylamide gel electrophoresis using Coomassie staining.

### Error function fitting and determination of the point spread function

The intensity drop at the end of microtubules was analysed by performing a line scan along microtubules in ImageJ and fitting the resulting intensity profile to the complementary error function (Equation 1) by nonlinear least square fit in R3.1.1.


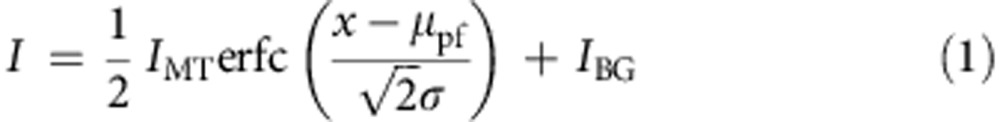


Here *I*_MT_, *μ*_pf_, *σ* and *I*_BG_ are microtubule intensity, mean protofilament length, tip s.d. (s.d._tip_) and background intensity, respectively. The point spread function for Cy5 dye in our microscope was determined by immobilizing Cy5 on coverslips through nonspecific binding, capturing an image and fitting to a two-dimensional Gaussian using FIESTA^59^. From the fits, sigma=131.4±4.3 nm (mean±s.e.m., *n*=17) corresponding to a full-width half-maximum of 309±10 nm.

To relate the s.d._tip_ from the error function to the degree of plus-end tapering (Fig. 2g), simulations were carried out as follows. The length of longest protofilament in a 13-protofilament microtubule was set to a fixed number of tubulin subunits, *N*_max_, the shortest protofilament was set to zero, and the lengths of the remaining 11 protofilaments were chosen randomly from a uniform distribution from 0 to *N*_max_. Tubulin subunits were labelled randomly using a dye density of 1 dye per 20 tubulin, matching experimental conditions, and one-dimensional intensity profiles were generated by convolving the position of each dye with a point spread function and fitting the resulting intensity profile with an error function (Fig. 2h). *N*_max_ was varied to establish the relationship between *N*_max_ and s.d._tip_ for the model, and this relationship was used to estimate the experimental tapering length (Fig. 2i).

## Additional information

**How to cite this article:** Chen, Y. & Hancock, W. O. Kinesin-5 is a microtubule polymerase. *Nat. Commun.* 6:8160 doi: 10.1038/ncomms9160 (2015).

## Supplementary Material

Supplementary Information Supplementary Figures 1-3 and Supplementary Table 1

Supplementary Movie 1 Microtubule growth in presence of kinesin-1. Experiment was carried out at 32°C with 10 μM unlabeled tubulin and 100 nM GFP-tagged kinesin-1. Scale bar is 2 μm.

Supplementary Movie 2 Microtubule growing with a bifurcated plus-end. Microtubules were polymerized in 10 μM unlabeled tubulin and 20 nM kin5_18 at 32°C. The binding of the GFP-labeled kin5_18 is sufficient to highlight the entire microtubules. Scale bar is 2 μm.

Supplementary Movie 3 Microtubule plus-end curling and breaking during elongation. Experimental conditions were the same as in Supplementary Movie 2. Scale bar is 1 μm.
