# Supplementary material for: Kinesin-5 is a microtubule polymerase
Source: Nat Commun. 2015 Oct 6;6:8160. doi: 10.1038/ncomms9160 (PMC4600729; doi:10.1038/ncomms9160)
Supplement: Supplementary Information — Supplementary Figures 1-3 and Supplementary Table 1 [file ncomms9160-s1.pdf]

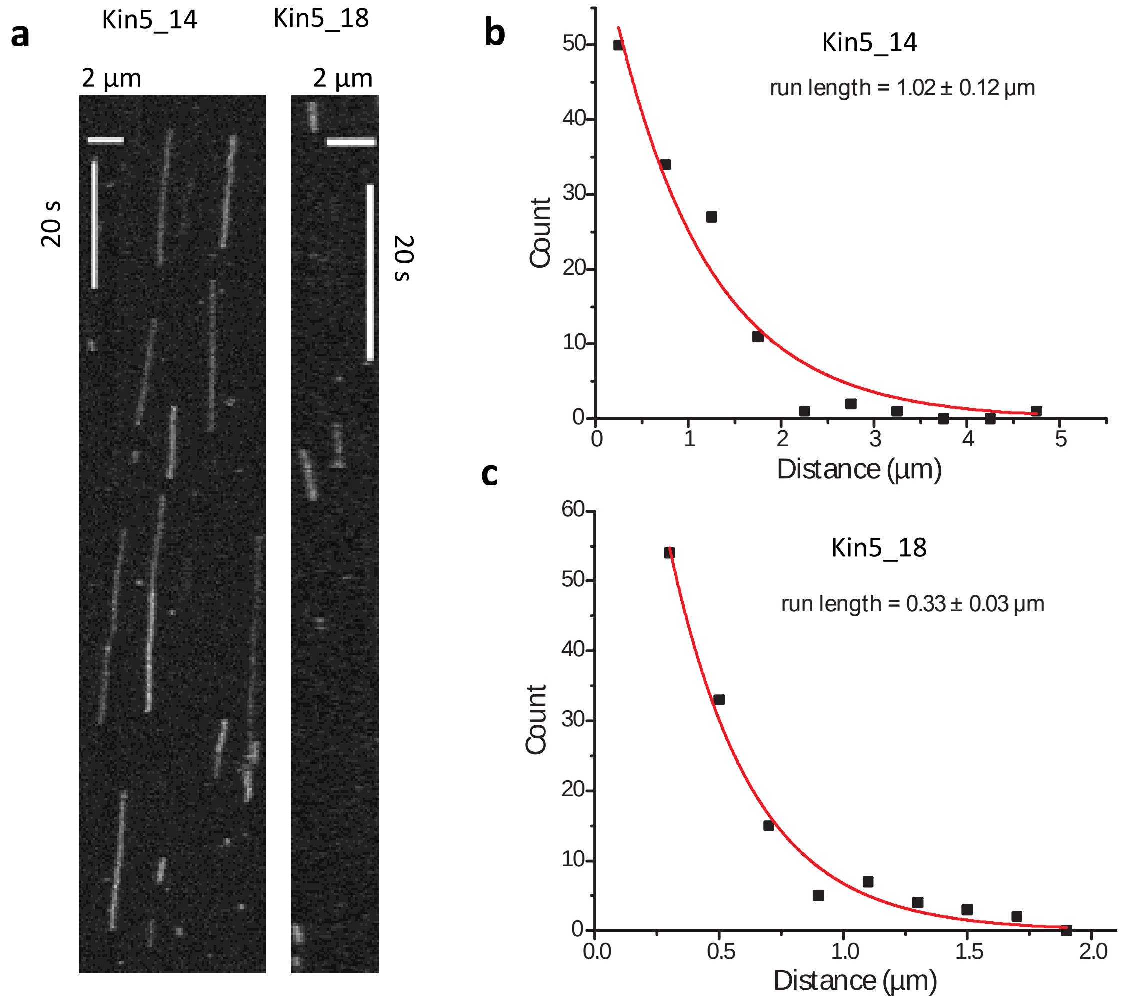

Supplementary Figure 1: Run length of kinesin-5. (**a**) Kymograph of kin5_14 (left) and kin5_18 (right). (**b**, **c**) run length measurement of kin5_14 and kin5_18. The black squares are binned raw measurements and red lines are exponential fit. The horizontal scale bar is 2 µm and the vertical bar is 20 s.

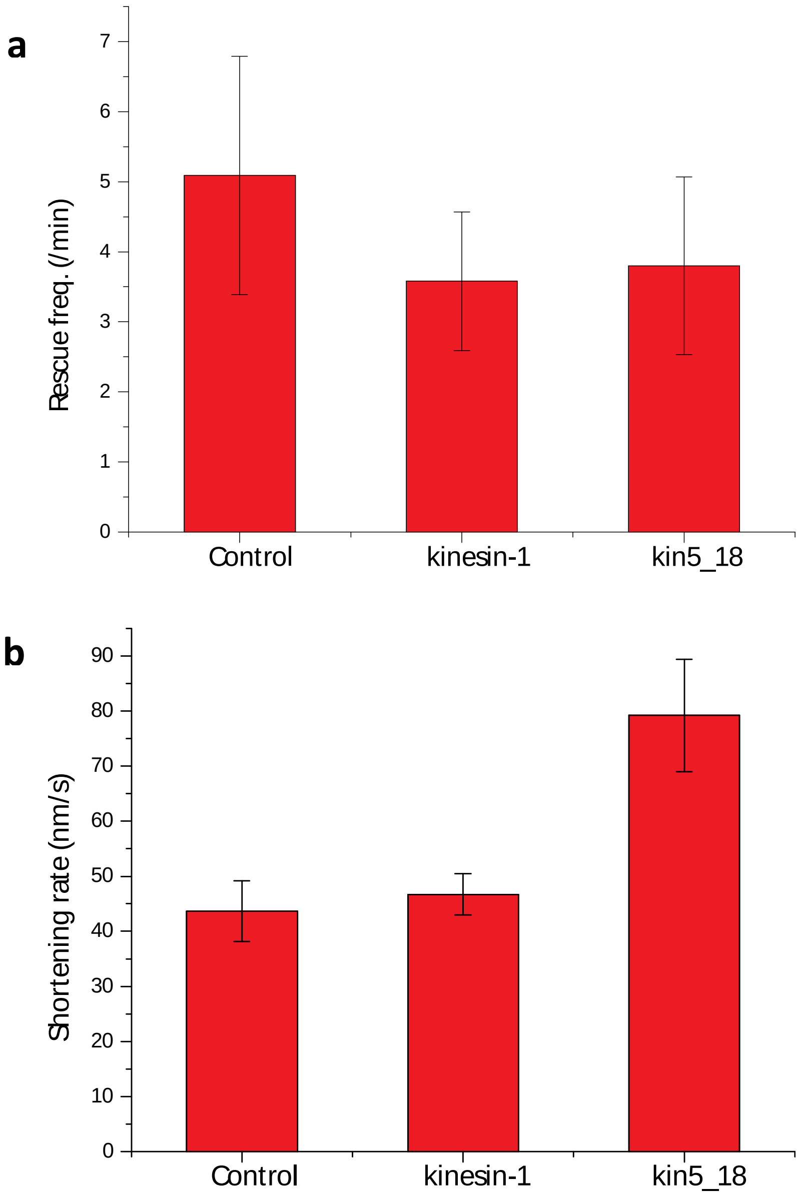

Supplementary Figure 2: **Rescue frequency and shortening rate of dynamic microtubules.** (**a**) Microtubules with kin5_18 showed slightly lower rescue frequency, but this difference is not statistically significant. (**b**) Shortening rate for microtubules with kin5_18 is significantly faster than control without motors.

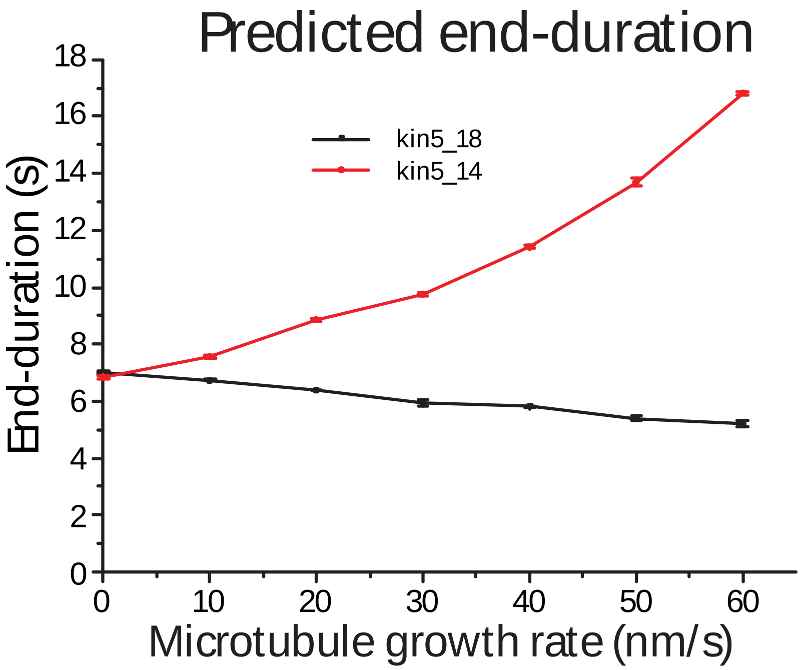

Supplementary Figure 3: Predicted end-duration of motors on dynamic microtubules. To relate processivity and end-binding to the observed +TIP-tracking activity, a model was developed in R@ 3.1.1 that simulated motor stepping on a growing microtubule. The model assumes the motor has a constant probability of detaching for every step that it takes, where p = 1/run length, and run length is expressed in number of 8 nm steps taken (128 for kin5_18 and 41 for kin5_14 based on Figure S1). In the absence of microtubule growth, motors pause at plus-ends for 7 seconds before detaching. If a tubulin subunit is added, the motor takes one 8 nm step (or detaches while taking that step) and the wait at the plus-end resets. For each microtubule growth rate, the duration that the motor tracked the growing plus-end was calculated. Because the duration of each processive run of Kin5_14 (17 s) is longer than the 7 second waiting time when reaching the end, the duration of end-binding for Kin5_14 is predicted to go up with increasing microtubule growth rates. Due to its shorter run duration (5.5 s), the end-duration of kin5_18 on dynamic microtubules is predicted to decrease with increasing microtubule growth rates. The finding that the end-duration of kin5_14 was not elevated on dynamic microtubules relative to stabilized microtubules suggests that a second motor dissociation pathway is involved, such as dissociation of the last tubulin at the plus-end with a motor attached. This second dissociation pathway may explain the lack observed end-binding by kin5_18 on dynamic microtubules.

	Growth rate (nm/s)	Catastrophe frequency (min^{-1})	**Length per growth phase (μm)**	Shortening rate (nm/s)	Rescue frequency (min^{-1})	**Length per shortening phase (μm)**	**Net growth rate (μm/min)**
Control	8.1	0.10	**4.9**	43.7	5.1	**0.51**	**0.43**
Kinesin-5	19.1	0.03	**38.2**	79.2	3.8	**1.25**	**1.10**

Supplementary Table 1: Effects of kinesin-5 on the net microtubule growth rate. Dynamic instability parameters presented in Figure 2B,C and S2 were used to calculate the mean length change during each growth or shortening phase. The mean duration of each growth phase is calculated as the inverse of the catastrophe frequency, and the mean duration of each shrinking phase is calculated as the inverse of the rescue frequency. Length changes are calculated as growth or shortening rate multiplied by time spent in that phase. Net microtubule growth rate is calculated as the net length change over one growth and shrinking cycle divided by the total duration of one growth and shrinking cycle. The enhanced growth rate and suppressed catastrophe frequency in the presence of kinesin-5 lead to an 8-fold increase in the length change during each growth phase. Thus, even though there is a 3-fold increase in the length of microtubule lost during each shortening event, the dynamics of the shortening phase have a minimal effect on the net microtubule growth rate.
